# Association between cognitive and autonomic dysfunctions in patients with de novo Parkinson’s disease

**DOI:** 10.1038/s41598-025-98508-9

**Published:** 2025-04-19

**Authors:** Byung-Euk Joo, Jihwan You, Rae On Kim, Kyum-Yil Kwon

**Affiliations:** Department of Neurology, Soonchunhyang University Seoul Hospital, 59 Daesagwan-ro, Yongsan-gu, Seoul, 04401 Republic of Korea

**Keywords:** Cognition, Dysautonomia, Mild cognitive impairment, Neurodegenerative disease, Parkinson’s disease, Neuroscience, Neurology

## Abstract

Cognitive decline is a common issue in Parkinson’s disease (PD) and significantly affects patients’ quality of life. This study explored the relationship between cognitive functions and dysautonomia in de novo PD. We reviewed records of newly diagnosed PD patients from July 2017 to September 2023 who underwent cognitive and autonomic assessments. Cognitive functions were measured using the Korean version of the Montreal Cognitive Assessment (MoCA-K) and the Seoul Neuropsychological Screening Battery, while autonomic functions were evaluated with the SCOPA-AUT questionnaire. Among 155 patients, 82 with de novo PD were included. The mild cognitive impairment (MCI) group exhibited higher SCOPA-AUT scores, particularly in gastrointestinal dysfunction. Multivariable logistic regression identified total SCOPA-AUT scores as significant predictors of MCI, even after adjusting for demographic and clinical factors. Partial correlation analysis showed significant negative associations between SCOPA-AUT scores and cognitive functions, such as memory and executive function. This study highlights a strong link between autonomic dysfunction, including gastrointestinal issues, and cognitive impairment in de novo PD. Monitoring dysautonomia in early-stage PD may aid in identifying patients at risk of cognitive decline.

## Introduction

Cognitive decline in Parkinson’s disease (PD) is not uncommon and tends to progressively worsen as the disease progresses. Particularly, the developing of dementia significantly impacts the quality of life of patients with PD. The prevalence of mild cognitive impairment (MCI) in patients with PD varies, with reports indicating a range of approximately 30% to 60%^1,2^. It is known that up to 83% of these patients may develop dementia 20 years after the onset of the disease^3^. A meta-analysis study reported that factors associated with cognitive decline or dementia in patients with Parkinson’s disease include older age, lower level of education, longer disease duration, more severe motor or axial symptoms, antiparkinsonian medication, apathy, and depression^1^. If clinicians carefully observe the clinical features of patients with PD, they can identify those exhibiting cognitive decline at an earlier stage, facilitating timely intervention. However, research on the clinical features related to cognitive decline in newly diagnosed PD patients remains unclear, indicating a need for further studies.

Research investigating the relationship between dysautonomia and MCI or dementia in PD has continued^4^. Especially, cardiovascular dysfunction is known to affect MCI in various stages of people with PD, including those with de novo PD^5,6^. Additionally, recent studies reported that gastrointestinal dysfunction or constipation is associated with MCI in patients with de novo PD^7–9^. Various cognitive dysfunctions and autonomic disturbances exist, yet the detailed relationships between impairments in different cognitive domains and various autonomic dysfunctions in PD have not been well understood. Therefore, in this study, we aimed to investigate the association between various autonomic dysfunctions and cognitive impairment in de novo PD patients. Moreover, we took a detailed approach to assessing cognitive impairment. In addition to examining autonomic dysfunction by comparing the MCI and non-MCI groups, we aimed to identify the relationships between global cognition, five cognitive domains, and various domains of autonomic function.

## Materials and methods

### Study design

This study was conducted as a retrospective cohort study, analyzing the medical records of patients from our hospital. It was approved by Soonchunhyang Institutional Review Board (IRB) (approval number: 2023-07-003) and conducted in accordance with the Declaration of Helsinki. Informed consent was waived by the Soonchunhyang IRB due to the study design.

### Participants

We reviewed the medical records of newly diagnosed Parkinson’s disease patients registered in our hospital’s Parkinson’s disease registry from July 2017 to September 2023. The clinical diagnosis of Parkinson’s disease followed the UK Brain Bank criteria, and only patients with confirmed typical dopaminergic loss on dopaminergic imaging were included to ensure diagnostic accuracy. To achieve the objectives of this study, we restricted the analysis to patients who underwent detailed cognitive assessments using both MoCA-K^10^ and the Seoul Neuropsychological Screening Battery, 2nd Edition (SNSB-II)^11^, and autonomic function assessments using the Korean version of the Scale for Outcomes in Parkinson’s disease-Autonomic (SCOPA-AUT) questionnaire^12^. Patients with atypical clinical features, such as dementia within the first year, recurrent falls, severe orthostatic hypotension or urinary incontinence, and supranuclear gaze palsy, were excluded. Additionally, patients with significant structural lesions on brain MRI, including considerable white matter changes or stroke, were excluded. Out of the 155 patients registered in the registry, 82 patients were finally included for this study; out of a total of 155 patients presenting with parkinsonism, 37 were not in the de novo state at the time of evaluation, 13 were identified as having atypical or unspecified parkinsonism, 12 were identified as having secondary parkinsonism including drug-induced parkinsonism, and 11 were unable to undergo the SNSB-II test.

### Clinical evaluations

At the initial visit, we assessed motor symptoms of patients by checking the UPDRS part 3 and Hoehn and Yahr stage in their drug-naive state, along with collecting baseline demographic data^13^. For dysautonomia, we investigated various autonomic dysfunctions using SCOPA-AUT, one of the highly recommended scales by the Movement disorders society (MDS)^14^. Cognitive function was thoroughly examined using MoCA-K and SNSB-2. Specifically, SNSB-2 conducted multiple cognitive tests across five cognitive domains. For each cognitive domain, we selected two representative tests and applied the cutoff value of 1.5 standard deviations according to the MDS PD-MCI criteria to identify patients with MCI^15^. Attention was assessed using the Digit Span Test forward and backward. Language was evaluated with the Korean version of the Boston Naming Test (K-BNT) and the animal component of the Controlled Oral Word Association Test (COWAT). Visuospatial function was measured using the Rey Complex Figure test (RCFT) copying test and the clock drawing test. Memory was checked with the Seoul Verbal Learning Test (SVLT)—delayed recall and the RCFT—delayed recall. Frontal executive function was assessed using the phonemic component of the COWAT and the Stroop test—color reading.

### Statistics

To compare the two groups, we employed the chi-square test for categorical variables and either Student’s t-test or the Mann–Whitney U test for continuous variables. To predict specific autonomic dysfunctions associated with MCI in patients with PD, we conducted logistic regression analysis. Multivariable logistic regression was carried out using a stepwise selection method, incorporating variables from the univariable logistic regression analysis and adjusting for age, sex, education level, PD duration, UPDRS-III score, and HY stage. To examine the relationship between individual cognitive tests and SCOPA-AUT scores, we performed partial correlation analysis. Statistical significance was defined as *p* < 0.05. All statistical analyses were conducted using Rex version 3.6.3 (URL: http://rexsoft.org).

## Results

### Demographic and clinical characteristics of patients with de novo Parkinson’s disease

The demographic and clinical characteristics of 82 patients with de novo PD were presented in Table 1. The mean age of participants was 70.30 ± 9.31 years and 56.10% of them were female. The mean duration of the disease was 1.39 ± 0.97 years. The mean UPDRS-Part III (motor) score was 23.89 ± 11.42), and the median modified Hoehn and Yahr (HY) stage was 2.0, with a range of 1 to 3.

**Table 1:** Demographic and clinical characteristics of patients with de novo Parkinson’s disease.

Variable	N = 82
Age, yr	70.30 ± 9.31
Sex-female	46 (56.10%)
Level of education, yr	9.63 ± 4.90
Disease duration, yr	1.39 ± 0.97
UPDRS-Part III (motor)	23.89 ± 11.42
The HY stage	2.0 [1–3]
SCOPA-AUT, total score*	12.04 ± 8.22
Gastrointestinal score	2 [0–16]
Urinary score	6 [0–18]
Cardiovascular score	0 [0–6]
Thermoregulatory score	0 [0–6]
Pupillomotor score	0 [0–6]
MoCA-K score	23.52 ± 4.45
*SNSB-II*	
Attention and working memory	
Forward Digit Span, z-score	1.64 ± 0.79
Backward Digit Span, z-score	0.37 ± 1.23
Language	
Korean-Boston Naming Test, z-score	0.10 ± 1.17
COWAT animal, z-score	− 0.37 ± 1.07
Visuospatial function	
RCFT copying test, z-score	− 0.14 ± 1.11
Clock drawing test, normal	64 (78.05%)
Memory	
SVLT delayed recall, z-score	− 0.68 ± 0.99
RCFT delayed recall, z-score	− 0.34 ± 1.08
Frontal executive function	
COWAT phonemic, z-score	− 0.58 ± 1.12
Stroop test (color reading), z-score	− 0.39 ± 1.26

Numerical data are presented as mean ± SD or median [min–max] after conducting test of normality with Kolmogorov–Smirnov test. Non-numerical data are shown as number (%).

*Total score of SCOPA-AUT was calculated except for sexual domain, since only 25 patients answered the question for the sexual dysfunction.

UPDRS, Unified Parkinson Disease Rating Scale; HY, Hoehn and Yahr; SCOPA-AUT, Scales for Outcomes in Parkinson’s disease—Autonomic; MoCA-K, Korean version of Montreal Cognitive Assessment; SNSB, Seoul Neuropsychological Screening Battery; COWAT, Controlled Oral Word Association Test; RCFT, Rey Complex Figure Test; SVLT, SVLT, Seoul Verbal Learning Test.

The SCOPA-AUT total score was 12.04 ± 8.22. The MoCA-K score averaged 23.52 ± 4.45. Various domains of the SNSB-II were evaluated, including attention and working memory (Forward Digit Span z-score: 1.64 ± 0.79; Backward Digit Span z-score: 0.37 ± 1.23), language (Korean-Boston Naming Test z-score: 0.10 ± 1.17; COWAT animal z-score: − 0.37 ± 1.07), visuospatial function (RCFT copying test z-score: − 0.14 ± 1.11; Clock drawing test normal: 78.05%), memory (SVLT delayed recall z-score: − 0.68 ± 0.99; RCFT delayed recall z-score: − 0.34 ± 1.08), and frontal executive function (COWAT phonemic z-score: − 0.58 ± 1.12; Stroop test color reading z-score: − 0.39 ± 1.26).

### Comparison of clinical features in patients with de novo Parkinson’s disease with or without mild cognitive impairment

We compared the clinical features of patients with de novo Parkinson’s disease with mild cognitive impairment (MCI, n = 21) and without MCI (non-MCI, n = 61) in Table 2. Comparing the clinical features of patients with de novo PD between MCI and non-MCI showed no significant differences in demographic and motor symptoms. However, we found some differences in regards to autonomic dysfunction of SCOPA-AUT; The total dysautonomia score was also higher in the MCI group (15.86 ± 8.03) compared to the non-MCI group (10.72 ± 7.93, *p* = 0.016). Especially, the gastrointestinal score was significantly higher in the MCI group (median 4 [1–7]) compared to the non-MCI group (median 1 [0–4], *p* = 0.0319).

**Table 2:** Comparison of clinical features in patients with de novo Parkinson’s disease with or without mild cognitive impairment.

Variable	MCI (n = 21)	Non-MCI (n = 61)	*P* value
Age, yr	71.05 ± 8.67	70.05 ± 9.57	0.6604
Gender-female	10 (47.62%)	36 (59.02%)	0.4472
Level of education, yr	10.26 ± 4.55	9.41 ± 5.04	0.476
Disease duration, yr	1.50 ± 1.04	1.35 ± 0.94	0.5749
UPDRS-Part III (motor)	25.00 ± 13.51	23.51 ± 10.72	0.6497
The HY stage	2 [2 – 2.5]	2 [2–2]	0.416
SCOPA-AUT			
Gastrointestinal score	4 [1–7]	1 [0–4]	**0.0319**
Urinary score	9 [6–10]	5 [3–10]	0.092
Cardiovascular score	0 [0–1]	0 [0–1]	0.285
Thermoregulatory score	1 [0–2]	0 [0–1]	0.0695
Pupillomotor score	0 [0–1]	0 [0–0]	0.3252
Total dysautonomia score*	15.86 ± 8.03	10.72 ± 7.93	**0.016**

Numerical data are presented as mean ± SD for normal distribution or otherwise median (interquartile range) after conducting test of normality with Kolmogorov–Smirnov test. Non-numerical data are shown as number (%).

*Total score of SCOPA-AUT was calculated except for sexual domain, since only 25 patients answered the question for the sexual dysfunction.

UPDRS, Unified Parkinson Disease Rating Scale; HY, Hoehn and Yahr; SCOPA-AUT, Scales for Outcomes in Parkinson’s disease – Autonomic.

Significant values are in bold.

### Logistic regression analysis of SCOPA-AUT for MCI in patients with de novo PD

Through logistic regression analysis, we identified significant predictors of SCOPA-AUT for MCI in patients with de novo PD in Table 3. Univariable analysis indicated that the gastrointestinal score had an odds ratio (OR) of 0.1651 (95% CI 0.0194–0.3108, *p* = 0.0264). The total dysautonomia score showed an OR of 0.0748 (95% CI 0.0134–0.4604, *p* = 0.0169). In the multivariable analysis, after adjusting for age, sex, disease duration, level of education, UPDRS part III score, and HY stage, the total dysautonomia score remained significant with an OR of 0.1465 (95% CI 0.0282–0.2649, *p* = 0.0153).

**Table 3:** Logistic regression analysis of SCOPA-AUT for mild cognitive impairment in patients with de novo Parkinson’s disease.

	Univariable			Multivariable		
Variable	OR	95% CI	*p-*value	OR*	95% CI	*p-*value
Gastrointestinal score	0.1651	0.0194–0.3108	**0.0264**	–	–	–
Urinary score	0.0709	− 0.0327–0.1745	0.1800	− 0.1564	− 0.3695–0.0568	0.1504
Cardiovascular score	0.2559	− 0.1347–0.6465	0.1991	–	–	–
Thermoregulatory score	0.3068	− 0.0517–0.6652	0.0935	–	–	–
Pupillomotor score	0.1674	− 0.1255–0.9984	0.2626	–	–	–
Total dysautonomia score*	0.0748	0.0134–0.4604	**0.0169**	0.1465	0.0282–0.2649	**0.0153**

*Adjusted for age, sex, disease duration, level of education, the UPDRS part III score, and the HY stage.

OR, odds ratio; CI, confidence interval; SCOPA-AUT, Scales for Outcomes in Parkinson’s disease—Autonomic; UPDRS, Unified Parkinson Disease Rating Scale; HY, Hoehn and Yahr.

Significant values are in bold.

### Correlation between cognitive functions and dysautonomia in patients with de novo PD

**Fig. 1:**
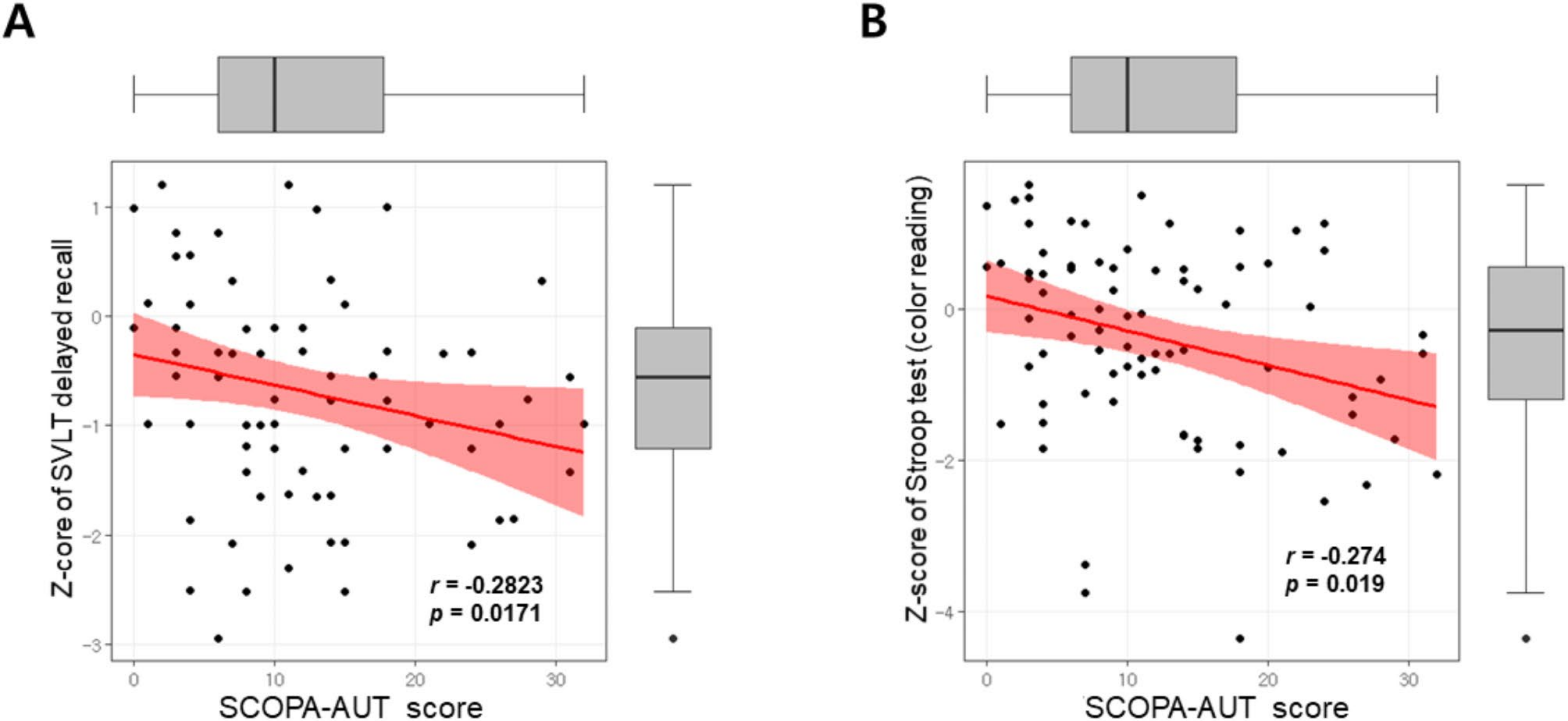
Correlation between dysautonomia and delayed verbal memory or frontal executive function of the Stroop color reading test. Negative correlations between the SCOPA-AUT total score and memory function of the SVLT delayed recall (Spearman’s r = − 0.2823, *p* = 0.0171) (**A**), and executive function of the Stroop test color reading (Spearman’s r = − 0.274, *p* = 0.019) (**B**).

We performed a partial correlation analysis to explore the relationship between various cognitive tests and SCOPA-AUT scores in patients with de novo PD in Table 4. The total SCOPA-AUT score was not significantly correlated with global cognition as measured by MoCA-K (Spearman’s r = − 0.0954, *p* = 0.4125). Among five cognitive domains, we revealed significant negative correlations between the SCOPA-AUT total score and memory function of the SVLT delayed recall (Spearman’s r = − 0.2823, *p* = 0.0171) (Fig. 1A), and executive function of the Stroop test color reading (Spearman’s r = − 0.274, *p* = 0.019) (Fig. 1B). Whereas, other cognitive domains did not show significant correlations with SCOPA-AUT scores.

**Table 4:** Partial correlation analysis of cognitive tests with SCOPA-AUT score in patients with de novo Parkinson’s disease.

Cognitive domain	Cognitive test	Total SCOPA-AUT score	
		Spearman’s r*	*p-*value
Global cognition	MoCA-K	− 0.0954	0.4125
Attention and working memory function	Forward Digit Span	0.0792	0.4992
	Backward Digit Span	0.0543	0.6437
Language function	Korean Boston Naming Test	− 0.1539	0.1845
	COWAT animal	− 0.0538	0.6445
Visuospatial function	RCFT copying test	− 0.1394	0.2298
Memory function	SVLT delayed recall	− 0.2823	**0.0171**
	RCFT delayed recall	0.216	0.0705
Executive function	COWAT phonemic	− 0.0191	0.8708
	Stroop test (color reading)	− 0.274	**0.019**

*Adjusted for age, sex, disease duration, level of education, the UPDRS part III score, and the HY stage.

SCOPA-AUT, Scales for Outcomes in Parkinson’s disease—Autonomic; MoCA-K, Korean version of Montreal Cognitive Assessment; COWAT, Controlled Oral Word Association Test; RCFT, Rey Complex Figure Test; SVLT, SVLT, Seoul Verbal Learning Test; UPDRS, Unified Parkinson Disease Rating Scale; HY, Hoehn and Yahr.

Significant values are in bold.

## Discussion

We investigated various autonomic symptoms using the SCOPA-AUT as well as detailed cognitive functions using the SNSB-II. The SCOPA-AUT is widely recommended by international movement disorders societies^12,14^. It could measure 25 items across six autonomic domains including gastrointestinal, urinary, cardiovascular, thermoregulatory, pupillomotor, and sexual part, making it a highly useful method for identifying a range of autonomic dysfunctions. However, in our study population, only 30% (25 out of 82) of participants responded to the sexual domains section, so this part of the analysis was excluded. This low response rate likely reflects the characteristics of the elderly population in our country, where many people do not have a spouse or do not engage in sexual activity. Additionally, in our country, the SNSB-II provides normative data for the general population, allowing us to calculate z-scores adjusted for sex, age, and education level for each cognitive function test^11^. In other words, the SNSB-II is a valuable tool for distinguishing MCI and conducting various analyses of cognitive functions.

When comparing the clinical features of de novo PD patients with and without MCI (Table 2), we found no significant differences in demographic factors or motor symptoms. However, there was a notable difference in SCOPA-AUT scores, with higher scores observed in the MCI group. This suggests a link between autonomic symptoms and cognitive decline in PD. Furthermore, our logistic regression analysis identified significant predictors of MCI related to autonomic dysfunction in de novo PD patients (Table 3). The total dysautonomia score emerged as a significant predictor, even after adjusting for age, sex, disease duration, education level, UPDRS part III score, and HY stage. This finding highlights autonomic dysfunction as a potential early marker for cognitive impairment in PD. These results suggest that targeting autonomic symptoms might have a beneficial impact on cognitive outcomes in patients with de novo PD.

As mentioned in the introduction, research on autonomic symptoms related to cognitive impairment in early stages of PD has primarily focused on specific autonomic domains, such as cardiovascular or gastrointestinal dysfunction^5–8^. Cardiovascular dysfunction, including orthostatic hypotension, is known to cause cerebral hypoperfusion, which can lead to cognitive impairment in PD^16^. On the other hand, gastrointestinal dysfunction, including constipation, is linked to the gut-brain axis, driven by interactions between the brain and gut microbiota. In other words, alterations in the gut microbiota could trigger the misfolding of α-synuclein, which may then propagate to the brain, contributing to the accumulation of Lewy bodies and the pathogenesis of PD^17,18^. Until now, there has been a lack of comprehensive studies examining the relationship between MCI and various autonomic functions, leaving this area insufficiently understood. Intriguingly, we can infer that gastrointestinal dysfunction, among various autonomic domains, may have a particularly strong association with MCI in patients with de novo PD (Tables 2 and 3). However, in the multivariable regression analysis, gastrointestinal dysfunction did not significantly predict MCI (the right panel, Table 3). This is likely due to collinearity, as the gastrointestinal domain score is a subscore of the SCOPA-AUT total score. Additionally, the sample size may have been too small for detailed analysis of each autonomic domain including gastrointestinal dysfunction. Especially, gastrointestinal symptoms are thought to be intricately associated with various mechanisms that can manifest in a range of symptoms across the upper and lower GI systems, including dyspepsia, gastroparesis, small bowel dysfunction, chronic constipation, and defecatory dysfunction^19^. Therefore, future research with a larger sample size and a more robust study design will be necessary to address these issues.

Cognitive decline in PD is known to be associated with complex functional neural networks, primarily involving frontostriatal and limbic regions. Additionally, it may be influenced by overlapping Alzheimer’s-related pathologies, potentially driven by interactions between α-synuclein, tau protein, and β-amyloid, alongside the core pathology of PD^20,21^. We identified a detailed relationship between cognitive functions and dysautonomia (Table 4 and Fig. 1). Dysautonomia assessed using SCOPA-AUT was not associated with global cognition measured by MoCA-K. However, SCOPA-AUT score showed negative correlations with only one item from the memory function tests (SVLT delayed recall) and one item from the frontal executive function tests (Stroop test color reading). Since our study explores the relationship between autonomic dysfunction and various cognitive functions in de novo PD, our findings suggest potential insights into the brain regions most vulnerable to autonomic dysfunction in the very early stages of the disease, including the hippocampus, other related structures in the temporal lobe, and the dorsolateral prefrontal cortex. Further research will be necessary to elucidate the precise pathomechanism underlying these findings.

The causes of cognitive decline in Parkinson’s disease are highly complex and remain an active area of research, particularly regarding the detailed pathophysiologic mechanisms and underlying factors. In particular, how autonomic dysfunction influences cognitive decline still requires substantial further research. Chung et al. previously reported a link between cognitive impairment and functional brain connectivity in both the fronto-subcortical and posterior cortical regions in patients with de novo PD who have dysautonomia^22^. Some researchers have suggested that cognitive decline might result from reduced brain perfusion and related cerebral ischemia caused by cardiovascular dysfunction^23^. On the other hand, beyond alterations in functional connectivity, neuropsychiatric manifestations such as apathy and depression can further complicate this relationship. Recent researches have shown that these neuropsychiatric symptoms in PD may contribute to or worsen autonomic dysfunction, as well as the dynamic interaction between cognitive and autonomic functions^24–27^. Future studies that incorporate these perspectives could offer valuable insights into how neuropsychiatric symptoms influence cognitive-autonomic interactions in PD, particularly in diverse cultural settings and in relation to their impact on daily life.

Our study has limitations. First, this study is a retrospective cross-sectional study conducted with a relatively small number of patients. Particularly, although we conducted a multivariable regression analysis, caution is needed when generalizing our results. Therefore, to confirm our findings, a well-designed follow-up study with a larger number of patients is necessary. Second, SCOPA-AUT is a useful test for assessing various autonomic functions, but since it relies on self-reported data, it can lack objectivity and introduce bias or inaccuracies. It’s also important to note that this study excluded questions about sexual dysfunction. Future research should include objective tests to verify various autonomic dysfunctions for more comprehensive results.

## Conclusion

In summary, our study highlights the significant relationship between autonomic dysfunction and cognitive impairment in de novo PD. Our data may suggest that identifying dysautonomia as a predictor of MCI provides a pathway for early detection and intervention. Especially, we could find that gastrointestinal dysfunction among various autonomic dysfunctions might be more associated with cognitive impairment in patients with de novo PD. Future research should aim to unravel the mechanisms behind these associations and develop strategies to mitigate cognitive decline by managing autonomic symptoms effectively.

## Data Availability

The datasets used and/or analysed during the current study are available from the corresponding author on reasonable request.
